# Mitogenome Characteristics and Phylogenetic Analysis of Six *Apistogramma* Species

**DOI:** 10.3390/ani16081178

**Published:** 2026-04-12

**Authors:** Xiao-Die Chen, Wei Hu, Xiao Ma, Cheng-He Sun, Chang-Hu Lu

**Affiliations:** College of Life Sciences, Nanjing Forestry University, Nanjing 210037, China; chenxiaodie5206@163.com (X.-D.C.); huwei@njfu.edu.cn (W.H.); 15578315324@163.com (X.M.)

**Keywords:** species identification, *Apistogramma*, mitogenome, molecular marker, phylogenetic analysis

## Abstract

The Neotropical cichlid genus *Apistogramma* represents one of the most taxonomically diverse and ecologically significant groups of South American freshwater fishes, yet its evolutionary history and species boundaries remain poorly understood due to a lack of comprehensive genomic resources. To address this gap, this study investigated the complete mitogenomic characteristics of six representative *Apistogramma* species (*A. agassizii*, *A. allpahuayo*, *A. baenschi*, *A. nijsseni*, *A. resticulosa*, and *A. cacatuoides*) to establish a robust molecular framework for species identification and phylogenetic reconstruction. The results showed that *Apistogramma* mitogenomes are highly conserved. All six *Apistogramma* species exhibited significant AT bias. Selection pressure analysis revealed that the Ka/Ks ratios for all 13 protein-coding genes were between 0 and 1, indicating that these genes were under purifying selection. Differential site analysis identified *nad5*, *cox1*, and *nad4* as ideal molecular markers for rapid *Apistogramma* species identification owing to higher proportions of variable sites. Phylogenetic analysis recovered *Apistogramma* as a strongly supported monophyletic clade (BP = 100, PP = 1.00), within which A. nijsseni clustered with *A. baenschi* and *A. cacatuoides* with *A. agassizii*. These internal phylogenetic relationships are consistent with the calculated genetic distances and previous morphological groupings. These findings provide an important theoretical basis and data support for rapid species identification, genetic evolutionary research, and divergence time estimation within *Apistogramma*.

## Simple Summary

We characterized the mitogenomes of six *Apistogramma* species, revealing highly conserved genomes with significant AT bias. All 13 protein-coding genes were under purifying selection. The genes *nad5*, *cox1*, and *nad4*, containing higher proportions of variable sites, could be ideal molecular markers for rapid species identification. Phylogenetic analysis confirmed the monophyly of *Apistogramma* and revealed closer relationships between *A. nijsseni* and *A. baenschi* and between *A. cacatuoides* and *A. agassizii*. These findings provide a basis for rapid species identification and evolutionary studies within this genus.

## 1. Introduction

Dwarf cichlids (Family: Cichlidae) represent a diverse assemblage of small-bodied fishes, typically defined by a maximum standard length of 10–12 cm. Widely distributed across South America and Africa, these teleosts are characterized by vibrant sexual dichromatism and complex behavioral traits [1,2]. Previous studies have highlighted their remarkable life-history strategies, particularly their rapid reproductive cycles and high adaptability to fluctuating freshwater environments [3]. While traditional research has extensively documented their ethology and morphology, recent molecular investigations have increasingly focused on resolving the complex phylogenetic patterns and cryptic diversity within species-rich genera [4]. However, genomic resources for many lineages remain limited, hindering a comprehensive understanding of their evolutionary history.

South American dwarf cichlids encompass 11 genera, including *Apistogramma* Regan 1913, *Dicrossus* Steindachner 1875, *Nannacara* Regan 1905, *Laetacara* Kullander 1986, and *Microgeophagus* Meulengracht-Madsen 1968. *Apistogramma*, the predominant genus, comprises over 100 species. Six representative species are particularly significant for phylogenetic and ecological studies: *A. agassizii* (Steindachner, 1875) (widely distributed across the Amazon, serving as a model for speciation); *A. trifasciata* (Eigenmann & Kennedy, 1903) (noted for its distinct sexual dimorphism); *A. borellii* (Regan, 1906) (exhibiting high tolerance for lower subtropical temperatures); and *A. cacatuoides* Hoedeman 1951 (frequently used in behavioral studies regarding harem-based social structures). *A. nijsseni* Kullander 1979 and *A. macmasteri* Kullander 1979 are also included, which represent distinct lineages within the Orinoco and Amazon drainages, respectively. Distributed across approximately two-thirds of South America, they primarily occupy the Amazon, Orinoco, and Paraná River basins, adapting to water conditions of pH 4.5–6.8 and temperature 26–28 °C. In contrast, West African dwarf cichlids, or West African kribs, constitute the genera *Pelvicachromis* Thys van den Audenaerde 1968 and *Nanochromis* Pellegrin 1904, which encompass a dozen varieties. Among them, *Pelvicachromis pulcher* (Boulenger 1901) is the most common and affordable species in the aquarium industry. High-end species commanding premium prices include *Pelvicachromis taeniatus* (Boulenger 1901), *Nanochromis nudiceps* (Boulenger 1899), and *Nanochromis transvestitus* Stewart & Roberts 1984. These demanding species require stringent environmental conditions and present considerable husbandry challenges for aquarists [5,6].

The fish exhibit mitochondrial DNA (mtDNA) sequences that are nearly identical to those of other vertebrates [7], containing no intronic sequences, except for a small segment related to mtDNA replication and transcription. Owing to their characteristics, including highly conserved coding regions, maternal inheritance, rapid evolutionary rate, and high copy number [8], mitochondrial genomes are widely applied in population genetics, biogeographical studies, and phylogeography [9,10]. Unlike shorter single-gene fragments, such as cytochrome c oxidase subunit I (*coxl*) and 12S ribosomal RNA (*12S rRNA*), which may reduce phylogenetic confidence due to uneven evolutionary rates and limited sequence length, complete mitochondrial genomes enable a more comprehensive phylogenetic reconstruction.

Neotropical cichlid fish exhibit extraordinary diversity; however, their morphological similarities and polymorphisms pose taxonomic challenges. This is particularly evident in the genus *Apistogramma*, which comprises more than 100 species with complex distribution patterns and unresolved chromosomal evolution mechanisms. South American *Apistogramma* species present substantial identification difficulties, necessitating the clarification of species boundaries, evolutionary relationships, and conservation status. Notably, female mate choice may drive sympatric speciation in this group. To address this, Quérouil et al. [11] developed highly polymorphic microsatellite markers (screening 7567 candidate loci to establish 13 polymorphic markers), which were validated across 47 specimens of 9 species for evolutionary genetics and conservation studies. Cytogenetic analyses by Wagner et al. [12] revealed that this genus possesses unique karyotypic features, including reduced chromosome numbers and an increased number of acrocentric chromosomes. Significant karyotype reorganization (involving B chromosomes and rDNA dynamics) suggests that chromosomal changes may act as barriers to post-zygotic isolation in sympatric species. Tougard et al. [13] analyzed 31 species using mitochondrial and nuclear markers, confirming monophyly, with four clades corresponding to three morphological lineages. Molecular dating has traced its origin to the Eocene (~50 mya), with differentiation driven by marine incursions. Although the Quaternary glaciation triggered speciation events, the genus maintains a persistently low and constant diversification rate.

Despite the ecological and commercial importance of *Apistogramma*, genomic resources for this genus remain notably deficient. While none of the species studied here are currently listed in the CITES Appendices, and most are categorized as ‘Least Concern’ (LC) or ‘Not Evaluated’ (NE) by the IUCN, their wild populations face mounting pressure from habitat degradation and over-collection for the aquarium trade [1,2]. Consequently, there is an urgent need for high-resolution molecular markers to monitor these genetic resources. Previous mitogenomic research has been largely restricted to isolated species descriptions, which often suffer from inadequate taxon sampling and low phylogenetic reliability. This fragmentation has hindered systematic comparative analyses of genomic architecture, such as nucleotide bias, codon usage, and potential gene rearrangement events. Leveraging the cost-effectiveness and efficiency of high-throughput sequencing (HTS) [14,15,16], this study performs a comprehensive genome survey—a recognized prerequisite for molecular research in non-model species [17]—to establish a robust phylogenomic framework and resolve the evolutionary history of *Apistogramma*.

In this study, we sequenced and characterized the complete mitochondrial genomes of six *Apistogramma* species (*A. agassizii* (Steindachner, 1875); *A. allpahuayo* Römer et al., 2012; *A. baenschi* Römer et al., 2004; *A. nijsseni* Kullander, 1979; *A. resticulosa* Kullander, 1980; and *A. cacatuoides* Hoedeman, 1951). Unlike previous studies that predominantly relied on short, single-gene fragments—which often lack sufficient phylogenetic signal to resolve the rapid radiation and cryptic diversity within *Apistogramma*—our mitogenomic approach provides an unprecedented level of resolution. By analyzing genome-wide structural features and utilizing multi-gene-concatenated datasets, we not only screened for high-resolution molecular markers for rapid species identification but also addressed long-standing taxonomic uncertainties and clarified the deep evolutionary relationships within the genus. This work establishes a robust molecular framework that facilitates a more accurate understanding of the diversification history of these Neotropical cichlids.

## 2. Materials and Methods

### 2.1. Sample Collection, Preservation, and Genomic DNA Extraction

All specimens were acquired as live individuals through the commercial ornamental fish trade from the Fangcun Flower, Bird, Fish, and Insect Market (23.06° N, 113.20° E), Liwan District, Guangzhou, China. Upon acquisition, the specimens were morphologically identified following the diagnostic keys. To ensure taxonomic accuracy, the identity of each species was further validated through subsequent mitogenomic sequencing and phylogenetic analysis in this study. The fish were anesthetized using an aquatic anesthetic (MS-222; tricaine mesylate) and immediately dissected. Dorsal muscle tissues were aseptically collected, flash-frozen in liquid nitrogen, transported to the laboratory, and stored at −80 °C until DNA extraction.

The specimen voucher numbers (stored at the Nanjing Forestry University Zoology Laboratory) and corresponding sequencing IDs for the five species sequenced de novo in this study are as follows: *A. resticulosa* (GDGZFC-Y36, PV692065), *A. nijsseni* (GDGZFC-Y37, PV739306), *A. baenschi* (GDGZFC-Y38, PV741065), *A. agassizii* (GDGZFC-Y39, PV747861), and *A. allpahuayo* (GDGZFC-S10, PV872131). Additionally, the complete mitochondrial genome sequence of *A. cacatuoides* was retrieved from the National Center for Biotechnology Information (NCBI) GenBank database (Accession No. KR150874) to facilitate comparative mitogenomic and phylogenetic analyses. For the five newly sampled species, total genomic DNA was extracted from approximately 30 mg of muscle tissue using the TIANamp Marine Animal DNA Kit (Tiangen Biotech, Beijing, China). The extraction followed the manufacturer’s optimized protocol for fibrous tissues, involving overnight lysis with proteinase K at 56 °C followed by purification via silica gel columns. The concentration and purity of the extracted DNA were quantified using a NanoDrop 2000 spectrophotometer (Thermo Scientific, Wilmington, DE, USA). Only samples with a ratio between 1.8 and 2.0 and a concentration exceeding 50 ng/μL were used for library construction. DNA integrity was further confirmed by 1% agarose gel electrophoresis, ensuring a sharp band of high-molecular-weight DNA without significant degradation [18].

Species were identified through a combination of morphological and molecular analyses. Morphological identification was performed by examining diagnostic characteristics, including caudal fin shape (rounded, truncate, lanceolate, or lyrate); the extension of dorsal fin spine membranes; and specific color patterns such as vertical stripes, lateral bands, and dots. A dichotomous key (see Table S1) was constructed and used according to published taxonomic revisions. For molecular validation, fragments of *rrnL*, *rrnS*, *cox1*, and *cytb* were sequenced. However, due to the paucity of species-specific sequences for *Apistogramma* in the NCBI database, molecular data were primarily utilized to confirm genus-level placement, with morphological characters serving as the primary diagnostic tool for species-level assignment.

### 2.2. Mitogenome Sequencing

DNA samples meeting quality standards were submitted to Shanghai Personal Biotechnology Co., Ltd. (Shanghai, China) for high-throughput sequencing. Qualified DNA samples were randomly fragmented, and paired-end libraries with an insert size of 500 bp were constructed using the Novogene NGS DNA Library Prep Kit (Novogene, Beijing, China). Following the manufacturer’s protocol, libraries were subjected to 150 bp paired-end sequencing on the Illumina NovaSeq platform (Illumina, San Diego, CA, USA) at Novogene (Beijing, China). Raw sequences were subjected to quality trimming and filtering with Trimmomatic v0.39 (http://www.usadellab.org/cms/index.php?page=trimmomatic, accessed on 9 April 2026) to generate high-quality clean data.

### 2.3. Mitogenome Assembly and Annotation

Clean sequencing data were assembled using GetOrganelle v1.7.1a (https://github.com/Kinggerm/GetOrganelle, accessed on 9 April 2026) [19] to generate contigs and scaffolds. The assembled sequences were aligned against the sequences in the NCBI NT database (accessed on 9 April 2026) using BLASTn v2.13.0, with mitochondrial sequences extracted from each assembly. Gap closure was performed on contigs using GapFiller v2.1.1 (https://sourceforge.net/projects/gapfiller/, accessed on 9 April 2026), followed by sequence polishing with Pilon v1.23 to obtain the final mitochondrial genome sequences. Structural and functional annotations were performed using the web application MitoFish (https://mitofish.aori.u-tokyo.ac.jp/, accessed on 9 April 2026) [20].

### 2.4. Mitogenome Sequence Analysis

The mitochondrial genome sequence of *A. cacatuoides* was retrieved from GenBank (KR150874). For gene-specific analyses, protein-coding gene (PCG) alignment was performed. Briefly, 13 PCGs from six *Apistogramma* species were aligned using MAFFT 7.505 [21]. For codon usage analysis, relative synonymous codon usage (RSCU) was calculated for each amino acid using PhyloSuite 1.2.3 [22]. For selection pressure assessment, synonymous (Ks) and nonsynonymous (Ka) substitution rates were computed using KaKs_calculator 2.0 [23], applying the vertebrate mitochondrial genetic code. For comparative genomics analysis, DnaSP 6 [24] and MEGA X [25] were employed for polymorphism analysis of the 13 PCGs and 2 rRNA genes, with the variables analyzed comprising total sites, conserved sites, variable sites, singleton variable sites, parsimony-informative sites, and proportion of variable sites. Genetic distances (Dxy) and their standard errors (SEs) were calculated using the Kimura 2-parameter (K2P) model in MEGA X [25], with 1000 bootstrap replicates to estimate the SE for each pairwise comparison.

### 2.5. Phylogenetic Analysis

For concatenated dataset construction, the 13 PCGs were aligned individually and then concatenated using MEGA X [20]. The mitochondrial genome sequences of 42 New World cichlid species were retrieved from GenBank and combined with those of five newly sequenced and annotated *Apistogramma* species (Table 1), which were strategically selected based on a combination of taxonomic breadth, phylogenetic relevance, and data integrity. Specifically, we included representatives from all major tribes within the subfamily Cichlinae (e.g., Geophagini, Cichlasomatini, Heroini, and Astronotini) to ensure broad evolutionary coverage of Neotropical lineages. To enhance the resolution of intergenic relationships, priority was given to all available complete mitogenomes of closely related ‘Geophagines’ groups. Furthermore, a rigorous quality filter was applied, selecting only high-quality mitochondrial genomes that contained a complete set of 13 PCGs, thereby ensuring the reliability of the subsequent phylogenomic reconstruction. We employed the midpoint-rooting method to polarize the phylogeny, as this approach is widely used when a clear, closely related outgroup is either unavailable or may introduce long-branch attraction (LBA) artifacts [26]. The stability of this rooting was further verified by its consistency with previous higher-level molecular studies of New World cichlids [27,28]

The PCGs of all 47 New World cichlids were aligned using MAFFT 7.505 and optimized using MACSE. The optimized data were processed using Gblocks [29] to remove unreliable sequences. Gene concatenation was performed using the built-in pipeline of PhyloSuite 1.2.3 [22], and the optimal nucleotide substitution model was estimated using ModelFinder 2.2.0 [30]. The best-fit substitution models for each partition were selected using ModelFinder 2.2.0 [30] based on the Bayesian Information Criterion (BIC). For Bayesian inference (BI), the selected models were GTR+F+I+G4 for *atp6*, *atp8*, *cox1–3*, *cytb*, *nad1–3*, *nad4L*, and *nad5*; GTR+F+I+G4 for *nad4*; and HKY+F+I+G4 for *nad6*. For maximum likelihood (ML) analysis, the models included TVM+F+I+G4 for most PCGs and K3Pu+F+I+G4 for *nad6*. Evolutionary relationships of New World cichlids were reconstructed using both ML and BI methods; the ML analysis was executed in IQ-TREE 2.2.0 [31], with 50,000 bootstrap replicates (Ultrafast Bootstrap). The branch support was evaluated using bootstrap probability (BP). The BI analysis [32] employed two independent Markov Chain Monte Carlo approaches set to run a total of 100,000,000 generations. Sampling was performed once every 1000 generations. The first 25% of the samples were discarded as aging samples. The posterior probability (PP) was calculated according to the remaining samples, and the Bayesian PP value of each node was calculated. The final phylogenetic trees (ML and BI) were visualized with iTOL v6 [33].

## 3. Results

### 3.1. Mitogenome Characteristics

The mitochondrial genomes of the six *Apistogramma* species have a double-stranded circular structure, encoding 37 genes: 13 PCGs, 2 rRNAs, and 22 tRNAs. The genome lengths range from 16,767 to 17,439 bp, with *A. allpahuayo* possessing the largest genome and *A. baenschi* having the smallest (Table 1). These values are consistent with the mitogenome sizes previously reported for other Neotropical cichlids. Comparative genomic arrangement analysis revealed a highly conserved gene order across the 47 New World cichlid mitochondrial genomes. Nucleotide composition analysis (Table 1) demonstrated a universal AT bias across all 47 genomes; *Bujurquina mariae* (Eigenmann 1922) showed the highest AT content (58.8%), while *Crenicichla regani* (Ploeg 1989) (incomplete genome) has the lowest (51.6%). Base composition heterogeneity assessed via nucleotide skew indices (AT-skew and GC-skew) revealed positive AT-skew values (indicating higher A than T) in all species except *Andinoacara rivulatus* (Günther 1860), *A. allpahuayo*, *Dicrossus filamentosus* (Ladiges 1958), and *Taeniacara candidi* Myers 1935. Negative GC-skew values (indicating higher C than G) were observed in all species except *A. allpahuayo*.

### 3.2. PCG Analysis

**Figure 1:**
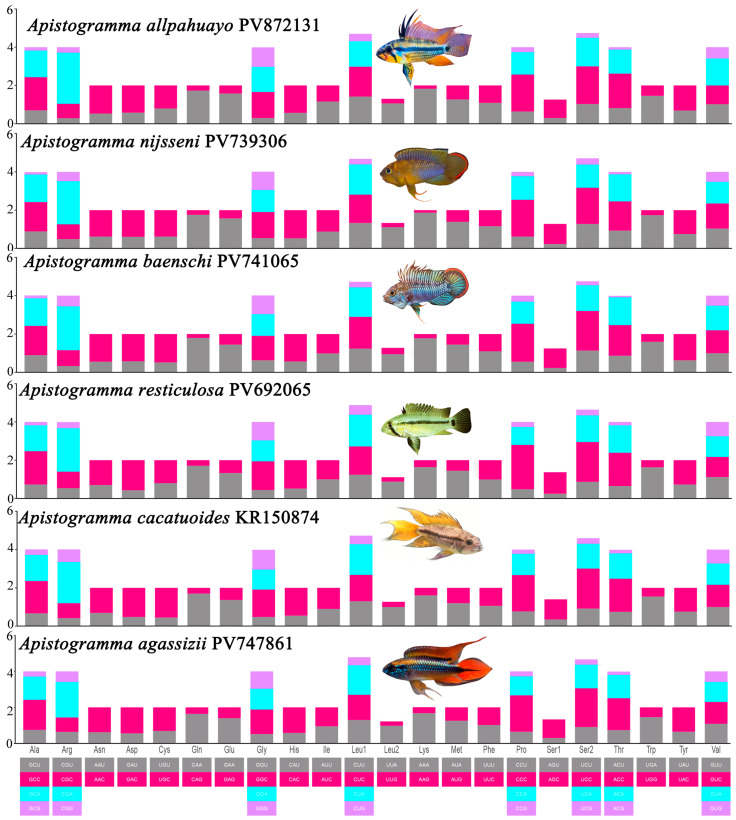
Relative synonymous codon usage (RSCU) distribution of the mitochondrial genome codons in six *Apistogramma* species.

**Figure 2:**
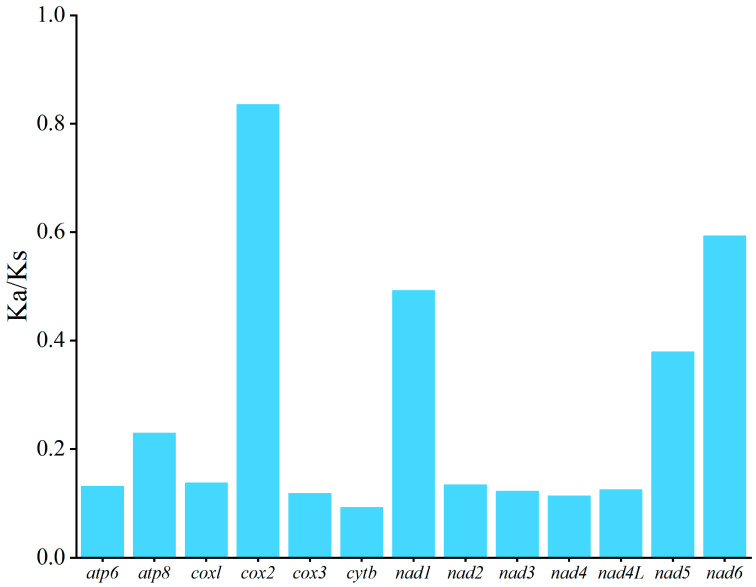
Selection pressure analysis of the mitochondrial genome in six *Apistogramma* species.

The RSCU analysis of the six *Apistogramma* mitochondrial genomes (Figure 1) revealed that all amino acids are encoded by two or more synonymous codons. Among the 60 vertebrate genetic codons (excluding stop codons), 24 high-frequency codons (RSCU > 1.00 in all six species) were identified. These codons exhibited a strong bias toward A or C at the third codon position: 12 codons ended with A, 11 codons ended with C, and only 1 codon ended with U. To assess evolutionary conservation across *Apistogramma* species, evolutionary rates of PCGs were evaluated using the Ka/Ks ratio (Figure 2). All 13 PCGs showed Ka/Ks < 1, indicating pervasive purifying selection. The evolutionary rates varied among the genes, with *cytb* exhibiting the strongest purifying selection (Ka/Ks = 0.091). Notably, *cox2* displayed a relatively high Ka/Ks ratio (0.934) compared to other PCGs. This elevated value suggests a relaxation of selective pressure or potential adaptive evolution within specific lineages of *Apistogramma*, a phenomenon that has been occasionally observed in rapidly diversifying cichlids. However, all genes remain under negative selection overall, maintaining the functional integrity of the mitochondrial respiratory chain.

### 3.3. Molecular Marker Screening and Evaluation

The nucleotide polymorphism analysis of mitochondrial genes across the six *Apistogramma* species revealed significant variations in gene conservation (Table 2). *rrnS* exhibited the lowest conservation (nucleotide variation rate: 19.50%), being the only gene with variation below 20.00%, and *nad6* showed the highest number of polymorphisms (variation rate: 50.28%). Sequence characteristics further revealed candidate molecular markers: *nad5* had the longest sequence (1848 bp), followed by *rrnL* (1733 bp), *cox1* (1573 bp), and *nad4* (1381 bp). The top three genes by variable sites included *nad5* (620 sites), *cox1* (526 sites), and *nad4* (450 sites). Given their high variation proportions, it was inferred that *nad5, cox1*, and *nad4* would be ideal molecular markers for the rapid identification of six *Apistogramma* species.

### 3.4. Phylogenetic Relationships

**Figure 3:**
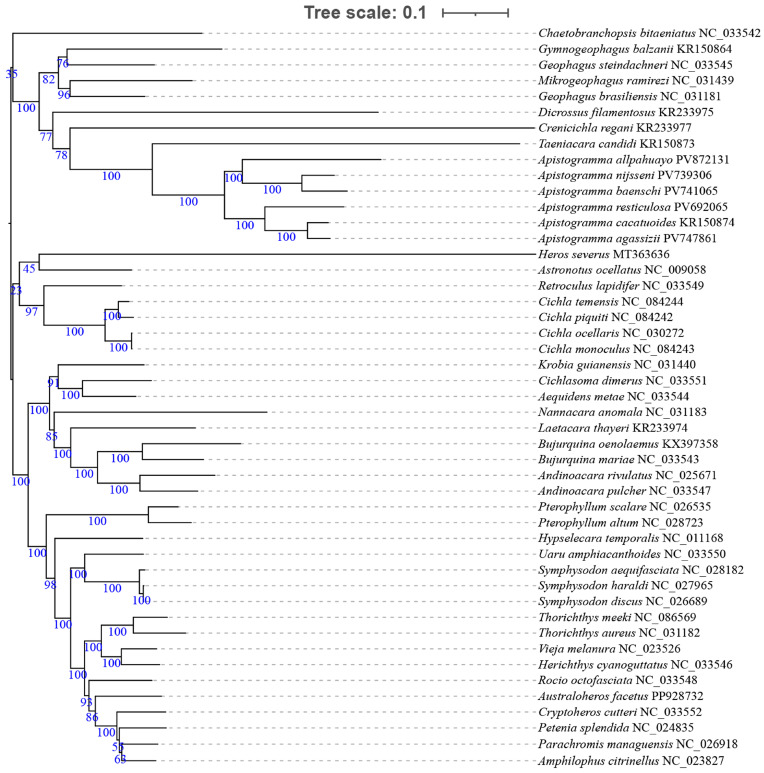
Maximum likelihood (ML) phylogenetic tree of 47 New World cichlid species based on the 13 PCG nucleotide sequences. The values on the branches represent bootstrap support, and the numbers following the species names are GenBank accession numbers. The ML tree is midpoint-rooted, with the root representing the hypothetical common ancestor of the studied taxa.

**Figure 4:**
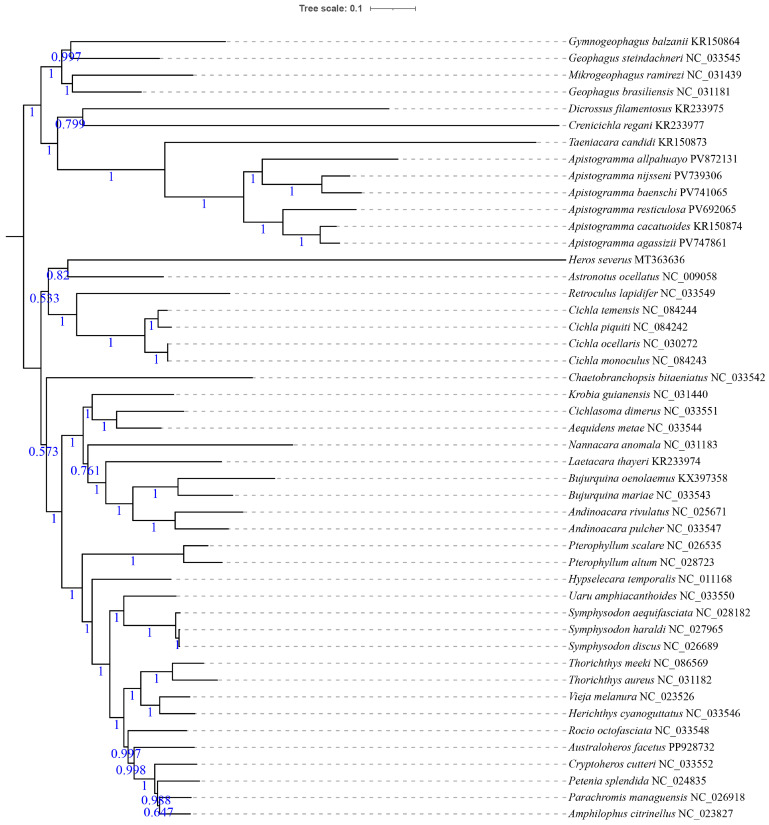
Bayesian inference (BI) phylogenetic tree of 47 New World cichlid species based on 13 PCG nucleotide sequences. The values on the branches represent posterior probabilities, and the numbers following the species names are GenBank accession numbers. The BI tree is midpoint-rooted, with the root representing the hypothetical common ancestor of the studied taxa.

Phylogenetic trees of New World cichlids were reconstructed based on the concatenated dataset of 13 PCGs using both BI and ML methods. The two topologies exhibited broad congruence (Figure 3 and Figure 4), with discordance observed only during the placement of *Chaetobranchopsis bitaeniatus* (Steindachner 1875). In our mitogenomic analysis, most major groups within the New World cichlids were recovered as non-monophyletic; specifically, both Cichlasomatinae and Geophaginae failed to form monophyletic clades in both BI and ML trees. This is evidenced by the placement of *Heros severus*, which was recovered outside the primary Cichlasomatinae lineage, and the division of Geophaginae into two distinct major clades. Similarly, Cichlinae and Astronotinae were also confirmed as non-monophyletic groups, highlighting the complex evolutionary history and potential mitonuclear discordance within Neotropical cichlids.

Among eight multi-species genera (≥2 species), only *Geophagus* Heckel 1840 was non-monophyletic. Monophyly was confirmed in *Apistogramma*, *Cichla* Bloch & Schneider 1801, *Andinoacara* Musilová, Říčan & Novák 2009, *Bujurquina* Kullander 1986, *Pterophyllum* Heckel 1840, *Symphysodon* Heckel 1840, and *Thorichthys* Meek 1904. Within *Apistogramma* (strongly monophyletic: BS = 100%, PP = 1), *A. nijsseni* and *A. baenschi* formed a clade, while *A. cacatuoides* and *A. agassizii* formed another clade, with the following observed relationships: (*A. allpahuayo* + (*A. nijsseni* + *A. baenschi*)) + (*A. resticulosa* + (*A. cacatuoides* + *A. agassizii*)).

### 3.5. Genetic Distance Analysis

Pairwise genetic distances (Dxy ± SE) based on the 13 concatenated PCGs revealed significant divergence among the six *Apistogramma* species (Table 3). The genetic distances ranged from 0.059 ± 0.002 (between *A. cacatuoides* and *A. agassizii*) to 0.233 ± 0.005 (between *A. allpahuayo* and *A. agassizii*). The observed genetic distances are highly congruent with the recovered phylogenetic topologies. The minimum distance (0.059) was identified between *A. cacatuoides* and *A. agassizii*, which formed a well-supported sister clade in our trees, suggesting a relatively recent speciation event. In contrast, *A. allpahuayo* exhibited consistently high divergence values (all > 0.219) when compared to the other five congeners. This high level of genetic differentiation (exceeding 20%) not only aligns with its basal/distinct position in the phylogenetic trees but also provides strong molecular evidence for its status as a distinct species, potentially representing a deeply diverged lineage within the genus.

## 4. Discussion

A comparative analysis of the mitogenomes of six *Apistogramma* species confirmed a conserved gene composition and arrangement consistent with those in most teleost fishes while also revealing nuanced features of their genomic evolutionary dynamics [34]. All six *Apistogramma* species displayed a moderate AT bias (51.6–58.8%), which is highly consistent with the typical range reported for teleost mitochondrial genomes (50–60%). This compositional bias is primarily attributed to strand-specific mutational pressure occurring during the asymmetric replication of mitochondrial DNA [35]. To ensure a robust estimation of evolutionary pressure, Ka/Ks ratios were calculated by accounting for common sequence-related biases following the methodological framework described by Del Amparo et al. [36]. Our results indicated strong purifying selection across all PCGs, underscoring the functional constraint of mitochondrial genes as core components of cellular energy production [37]. This pattern aligns with the “energetic functional constraint” hypothesis observed across teleost fishes, which suggests that the mitochondrial genome is under stringent selection to maintain high metabolic efficiency [38]. Notably, selective pressure varied among genes: *cytb, nad4,* and *cox3* exhibited very low Ka/Ks values. The extreme conservation of these genes likely reflects their indispensable roles in the assembly and electron-transfer efficiency of OXPHOS complexes (Complex III, IV, and I, respectively). Mutations in these core subunits could lead to mitochondrial dysfunction and significant fitness costs, a phenomenon also documented in other specialized fish groups like Salmonids, where maintaining oxidative capacity is vital for survival [39]. Compared to the adaptive evolution often identified in migratory or cold-adapted fishes (e.g., Salmonidae), the relatively uniform and low Ka/Ks ratios in *Apistogramma* suggest an evolutionary strategy focused on stabilizing the existing metabolic machinery within their tropical freshwater niches [40]. This study establishes a comparative mitogenomic framework for *Apistogramma*, providing a basis for evaluating interspecific genetic diversity.

*Apistogramma* is valuable in evolutionary and biogeographic research, where suitable molecular markers are essential for taxonomic precision. Although *cytb* and *cox1* have been the most frequently utilized traditional markers for species identification in this genus [13,41], their resolution is often limited when distinguishing closely related or recently diverged taxa. To address the limited resolution of these conventional markers, we systematically assessed variations across the complete mitogenome. Our screening revealed—for the first time—that *nad5* and *nad4* show higher proportions of interspecific variation than the conventional markers *cytb* and *cox1*. This finding suggests that incorporating *nad5* or *nad4* could substantially improve the identification of morphologically similar or recently diverged species within the genus. These results offer important data and new candidate markers for developing a high-resolution DNA barcoding system for *Apistogramma*, moving beyond reliance on single-gene approaches. The candidate markers will enable robust biodiversity assessment and support rapid species identification across the genus.

Phylogenetic trees reconstructed from 13 PCGs yielded largely congruent topologies with high statistical support, aligning with earlier studies [13] and confirming the close relationship between *A. nijsseni* and *A. baenschi*. From a molecular perspective (DNA influence), the transition from single-gene markers to complete mitogenomes significantly increased information density, providing the necessary resolution to distinguish these closely related taxa. However, these DNA-level divergences are intrinsically linked to ‘other influences,’ such as the complex hydrogeological history of the Amazon Basin [13]. For instance, the high genetic distance (Dxy > 0.2) and unique gene rearrangement observed in *A. allpahuayo* suggest a prolonged period of evolutionary isolation, potentially driven by vicariant events or river dynamics that restricted gene flow. Furthermore, the pervasive purifying selection (Ka/Ks < 1) identified across all PCGs reflects the functional constraints on DNA to maintain metabolic efficiency in varying ecological niches. While our mitogenomic framework establishes a robust maternal backbone, the potential for mitonuclear discordance—often caused by incomplete lineage sorting or ancient hybridization during rapid radiation—remains a factor. Therefore, these DNA-based findings provide a primary framework that integrates genomic architecture with the known biogeographic patterns of *Apistogramma*, offering a reliable foundation for future studies incorporating nuclear data and ecological parameters.

## 5. Conclusions

This study characterized the complete mitochondrial genomes of six *Apistogramma* species, revealing highly conserved genomic structures and universal purifying selection across all protein-coding genes. Our comparative analysis identifies *nad5*, *cox1*, and *nad4* as promising molecular markers that hold potential for rapid species identification, given their higher interspecific divergence compared to the traditional *cytb* gene. exhibit higher interspecific divergence than the traditional *cytb* gene. Furthermore, phylogenomic reconstruction confirms the monophyly of *Apistogramma* and clarifies key sister-species relationships, including those between *A. nijsseni*–*A. baenschi* and *A. cacatuoides*–*A. agassizii*. Future studies incorporating broader taxonomic sampling and intraspecific data are warranted to further validate the effectiveness of these markers and the presence of a distinct barcoding gap.

## Figures and Tables

**Table 1:** Basic characteristics of the mitogenome in *Apistogramma*.

Subfamily	Organism	ID	Full Length (bp)	A+T (%)	AT Skew	GC Skew
Astronotinae	*Astronotus ocellatus*	NC_009058	16,569	55.0	0.049	−0.342
Astronotinae	*Chaetobranchopsis bitaeniatus*	NC_033542	16,610	58.4	0.042	−0.351
Cichlasomatinae	*Aequidens metae*	NC_033544	16,541	53.6	0.037	−0.315
Cichlasomatinae	*Amphilophus citrinellus*	NC_023827	16,522	54.2	0.054	−0.340
Cichlasomatinae	*Andinoacara pulcher*	NC_033547	16,513	56.8	0.011	−0.299
Cichlasomatinae	*Andinoacara rivulatus*	NC_025671	16,585	56.9	−0.019	−0.259
Cichlasomatinae	*Australoheros facetus*	PP928732	16,556	54.6	0.036	−0.323
Cichlasomatinae	*Bujurquina mariae*	NC_033543	16,540	58.8	0.004	−0.286
Cichlasomatinae	*Bujurquina oenolaemus*	KX397358	16,532	57.5	0.012	−0.301
Cichlasomatinae	*Cichlasoma dimerus*	NC_033551	16,617	54.5	0.041	−0.327
Cichlasomatinae	*Cryptoheros cutteri*	NC_033552	16,528	52.9	0.040	−0.328
Cichlasomatinae	*Herichthys cyanoguttatus*	NC_033546	16,540	53.4	0.059	−0.344
Cichlasomatinae	*Heros severus*	MT363636	16,577	56.9	0.030	−0.221
Cichlasomatinae	*Hypselecara temporalis*	NC_011168	16,544	53.9	0.021	−0.316
Cichlasomatinae	*Krobia guianensis*	NC_031440	16,539	54.3	0.045	−0.324
Cichlasomatinae	*Laetacara thayeri*	KR233974	14,315	56.0	0.003	−0.303
Cichlasomatinae	*Nannacara anomala*	NC_031183	16,502	53.4	0.025	−0.301
Cichlasomatinae	*Parachromis managuensis*	NC_026918	16,526	53.6	0.049	−0.339
Cichlasomatinae	*Petenia splendida*	NC_024835	16,518	53.2	0.053	−0.338
Cichlasomatinae	*Pterophyllum altum*	NC_028723	16,495	54.2	0.014	−0.325
Cichlasomatinae	*Pterophyllum scalare*	NC_026535	16,491	54.2	0.016	−0.317
Cichlasomatinae	*Rocio octofasciata*	NC_033548	16,539	54.4	0.041	−0.340
Cichlasomatinae	*Symphysodon aequifasciata*	NC_028182	16,545	54.9	0.049	−0.335
Cichlasomatinae	*Symphysodon discus*	NC_026689	16,544	54.9	0.052	−0.337
Cichlasomatinae	*Symphysodon haraldi*	NC_027965	16,543	54.9	0.051	−0.336
Cichlasomatinae	*Thorichthys aureus*	NC_031182	16,530	52.1	0.042	−0.325
Cichlasomatinae	*Thorichthys meeki*	NC_086569	16,526	53.2	0.052	−0.339
Cichlasomatinae	*Uaru amphiacanthoides*	NC_033550	16,549	54.4	0.044	−0.326
Cichlasomatinae	*Vieja melanura*	NC_023526	16,543	52.6	0.058	−0.335
Cichlinae	*Cichla monoculus*	NC_084243	16,526	54.4	0.076	−0.350
Cichlinae	*Cichla ocellaris*	NC_030272	16,526	54.3	0.076	−0.350
Cichlinae	*Cichla piquiti*	NC_084242	16,536	54.3	0.079	−0.354
Cichlinae	*Cichla temensis*	NC_084244	16,530	54.1	0.087	−0.358
Cichlinae	*Crenicichla regani*	KR233977	11,461	51.6	0.030	−0.313
Geophaginae	*Apistogramma agassizii*	PV747861	16,941	54.3	0.025	−0.302
Geophaginae	*Apistogramma allpahuayo*	PV872131	17,439	55.5	−0.038	0.325
Geophaginae	*Apistogramma baenschi*	PV741065	16,767	55.0	0.055	−0.344
Geophaginae	*Apistogramma cacatuoides*	KR150874	16,870	54.3	0.025	−0.302
Geophaginae	*Apistogramma nijsseni*	PV739306	16,803	55.6	0.037	−0.326
Geophaginae	*Apistogramma resticulosa*	PV692065	16,909	54.0	0.040	−0.313
Geophaginae	*Dicrossus filamentosus*	KR233975	11,887	53.0	−0.002	−0.308
Geophaginae	*Geophagus brasiliensis*	NC_031181	16,559	54.1	0.044	−0.319
Geophaginae	*Geophagus steindachneri*	NC_033545	16,594	53.8	0.063	−0.339
Geophaginae	*Gymnogeophagus balzanii*	KR150864	16,587	56.0	0.018	−0.306
Geophaginae	*Mikrogeophagus ramirezi*	NC_031439	16,526	55.4	0.033	−0.294
Geophaginae	*Taeniacara candidi*	KR150873	16,581	57.2	−0.005	−0.290
Retroculinae	*Retroculus lapidifer*	NC_033549	16,537	52.8	0.058	−0.314

**Table 2:** Differential analysis of mitochondrial genes in six *Apistogramma* species.

Parameters	*atp6*	*atp8*	*coxl*	*cox2*	*cox3*	*cytb*	*nad1*	*nad2*	*nad3*	*nad4*	*nad4L*	*nad5*	*nad6*	*rrnL*	*rrnS*
Total number of sites	683	168	1573	691	783	1140	975	1045	346	1381	297	1848	531	1733	954
Invariable sites	438	95	1047	422	546	775	649	691	233	931	202	1228	264	1368	768
Variable sites	245	73	526	269	237	365	326	354	113	450	95	620	267	365	186
Singleton variable sites	113	45	218	169	129	181	163	168	50	208	38	296	108	205	109
Parsimony informative sites	132	28	308	100	108	184	163	186	63	242	57	324	159	160	77
Ratio of variable sites/%	35.87	43.45	33.44	38.93	30.27	32.02	33.44	33.88	32.66	32.59	31.99	33.55	50.28	21.06	19.50

**Table 3:** Pairwise genetic distances (Dxy ± SE) of the 13 mitochondrial protein-coding genes (PCGs) among species of the six *Apistogramma* species.

	*A. agassizii*	*A. allpahuayo*	*A. baenschi*	*A. cacatuoides*	*A. nijsseni*	*A. resticulosa*
*A. agassizii*						
*A. allpahuayo*	0.233 ± 0.005					
*A. baenschi*	0.214 ± 0.005	0.229 ± 0.005				
*A. cacatuoides*	0.059 ± 0.002	0.230 ± 0.006	0.215 ± 0.005			
*A. nijsseni*	0.211 ± 0.005	0.219 ± 0.005	0.098 ± 0.003	0.213 ± 0.005		
*A. resticulosa*	0.158 ± 0.004	0.240 ± 0.005	0.222 ± 0.005	0.155 ± 0.004	0.217 ± 0.005	

## Data Availability

The complete mitochondrial genome sequences and annotations are available in the National Center for Biotechnology Information (NCBI) GenBank database (https://www.ncbi.nlm.nih.gov/genbank/, accessed on 9 April 2026) under accession numbers PV747861, PV872131, PV741065, PV739306, and PV692065.

## Supplementary Materials

The following supporting information can be downloaded at: https://www.mdpi.com/article/10.3390/ani16081178/s1, Table S1: Identification Key for Six Common Species of the Genus *Apistogramma*. Refs. [42,43,44] are cited in the Supplementary Materials.

## Abbreviations

The following abbreviations are used in this manuscript:

BI	Bayesian Inference
BP	Bootstrap Probability
ML	Maximum Likelihood
mtDNA	Mitochondrial DNA
PCG	Protein-Coding Gene
PP	Posterior Probability
RSCU	Relative Synonymous Codon Usage
