# Supplementary material for: Mitogenome Characteristics and Phylogenetic Analysis of Six Apistogramma Species
Source: Animals (Basel). 2026 Apr 12;16(8):1178. doi: 10.3390/ani16081178 (PMC13112957; doi:10.3390/ani16081178)
Supplement: Supplementary file 1 [file animals-16-01178-s001.zip › animals-4240923-supplementary.pdf]

Table S1. Identification Key for Six Common Species of the Genus *Apistogramma*

Dichotomous Key

1. Caudal fin rounded or truncate , lacking extended tips or a lyrate shape... 2

   Caudal fin not rounded, appearing lanceolate or lyrate... 4

2. Caudal fin with distinct vertical dark stripes or a ladder-like dot pattern.. *A. resticulosa*

   Caudal fin without vertical stripes; margins usually possess red or orange bands (*nijsseni* group)............................ 3

3. Male dorsal fin anterior spine membranes (especially the 3rd–5th) significantly extended, forming a high serrated shape.......................................*A. baenschi*

   Male dorsal fin spine membranes not significantly extended; female lateral mid-spot is extremely large and round...*A. nijsseni*

4. Caudal fin lanceolate (spear-shaped), with the central portion pointed posteriorly... *A. agassizii*

   Caudal fin is lyrate, with upper and lower lobe tips extended and a concave central portion.. 5

5. Male dorsal fin 3rd to 5th (or 6th) spine membranes highly extended, crest-like; caudal fin usually covered with red and black variegated patches... *A. cacatuoides*

   Dorsal fin spine membranes without significant crest-like extension; lateral band significantly widened at the caudal peduncle, merging with a large caudal peduncle spot... *A. allpahuayo*

References

1. Kullander, S.O. A Taxonomical Study of the Genus Apistogramma Regan, with a Revision of Brazilian and Peruvian Species (Teleostei: Percoidei: Cichlidae); Bonner Zoologische Monographien; Zoologisches Forschungsinstitut und Museum Alexander Koenig: Bonn, Germany, 1980; Volume 14, 1–152.

2. Römer, U. Cichlid Atlas 1: Natural History of South American Dwarf Cichlids; Mergus Verlag: Melle, Germany, 2001.

3. Römer, U.; Beninde, J.; Duponchelle, F.; García-Dávila, C.R.; Díaz, A.C.; Hahn, I. Description of Apistogramma allpahuayo sp. n., a new dwarf cichlid species (Teleostei: Perciformes: Geophaginae) from in and around the Reserva Nacional Allpahuayo Mishana, Loreto, Peru. Vertebr. Zool. 2012, 62, 189–212.
